# Association Between Daily Food-Tracking Frequency and Clinically Significant Weight Loss: Retrospective Cohort Study of 5132 Mobile App Users

**DOI:** 10.2196/98144

**Published:** 2026-08-13

**Authors:** Sergey Oreshko, Susan Heikkinen

**Affiliations:** 1 MyNetDiary Inc, 621 NW 53rd St., Suite 240, Boca Raton, FL, 33487, United States, 1 8003857461

**Keywords:** dietary self-monitoring, weight management, mHealth, self-monitoring, mobile app, mobile health

## Abstract

In this retrospective cohort of 5132 users of a commercial nutrition-tracking mobile application, higher food-tracking frequency was associated with greater weight loss over 6 months; 70.1% (3599/5132) of users lost at least 5% of body weight.

## Introduction

Dietary self-monitoring supports weight management [1]. App-based dietary interventions can improve weight-related outcomes but supporting studies were generally small and often multicomponent [2]. Real-world evidence on tracking frequency is limited. We analyzed 6 months of MyNetDiary data to examine the association between food-tracking frequency and weight change in an unguided real-world cohort.

## Methods

We conducted a retrospective cohort analysis of users who signed up for the MyNetDiary mobile application (iOS and Android) during May 2025. Data were extracted by the authors, who were MyNetDiary Inc employees. From 412,193 new users, we applied filters: a weight-loss goal (n=341,962), 21 or more in-window food-logging days (n=51,087), weight recorded each month, June through November 2025 (n=5351), eligibility (age 18 y or older, recorded sex, plausible height, starting body mass index [BMI] of 18.5 or higher; n=5148), and exclusion of weight changes beyond 3 SDs of the cohort mean (−33.2% to +14.1%) as extreme outliers (n=5132).

The primary outcome was percentage body weight change over 6 months. Secondary outcomes were 5% or more and 10% or more loss [3]. The primary exposure was food-tracking frequency, defined as the average days per week with at least 1 food entry from signup through November 30, 2025, and categorized into 6 groups. Streak length, age group, sex, and starting BMI category served as secondary predictors. Multivariable logistic regression estimated the association between tracking frequency (continuous, days per week) and 5% or more loss, adjusted for age, sex, and starting BMI (n=5132). Sensitivity analyses used stricter tracked-day definitions, categorical frequency modeling, and a±15% outlier rule.

### Ethical Considerations

This retrospective analysis used deidentified app records without participant contact. The authors determined that it does not constitute human subjects research under 45 CFR 46.102(e)(1) [4]; institutional review board review and informed consent were not required. At registration, users accept a privacy policy permitting anonymous, aggregate research use of their data; study-specific consent was not sought. MyNetDiary Inc. maintains no institutional review board.

## Results

The cohort comprised 5132 users (mean age 42.1 y; mean starting weight 91.8 kg; 3736/5132, 72.8% women). Mean weight loss was 9.3 kg (9.5% of body weight). Overall, 70.1% (3599/5132) lost 5% or more, 44.8% (2299/5132) lost 10% or more, and 9.0% (460/5132) gained weight.

Weight loss increased across tracking-frequency categories (Table 1). Users tracking 6.0 to 7.0 days per week (2225/5132, 43.4%) lost 11.33% of body weight compared with 6.84% among users tracking fewer than 2.0 days per week. Each additional tracking day per week was associated with higher odds of 5% or more loss in the adjusted model (adjusted odds ratio, 1.35; 95% CI, 1.31‐1.40; P<.001; n=5132).

**Table 1.** Weight loss outcomes by tracking frequency (N=5132). Frequency categories are nonoverlapping; lower bounds are inclusive; 7.0 falls in the highest category. Baseline characteristics by category: Multimedia Appendix 1.

Tracking frequency	Users, n (%)	Mean % lost	5% or more lost, n (%)	Adjusted odds ratio (95% CI)
<2.0 days/wk	475 (9.3)	6.84	247 (52.0)	1 [Reference]
2.0 to <3.0 days/wk	480 (9.4)	7.35	271 (56.5)	1.19 (0.91‐1.55)
3.0 to <4.0 days/wk	540 (10.5)	7.77	323 (59.8)	1.41 (1.08‐1.82)
4.0 to <5.0 days/wk	642 (12.5)	7.95	410 (63.9)	1.80 (1.40‐2.31)
5.0 to <6.0 days/wk	770 (15.0)	9.82	569 (73.9)	2.95 (2.30‐3.80)
6.0 to 7.0 days/wk	2225 (43.4)	11.33	1779 (80.0)	4.35 (3.50‐5.42)

Stricter tracked-day definitions yielded adjusted odds ratios of 1.36, 1.36, and 1.27, and the ±15% outlier rule 1.32 (Multimedia Appendix 1). Longer consecutive-day streaks showed a similar pattern. Users with streaks exceeding 90 days (2021/5132, 39.4%) lost 11.55% of body weight, and 81.2% (1641/2021) achieved 5% or more loss, versus 4.21% and 42.3% (66/156), respectively, among users with streaks of 7 days or fewer (Welch *t* = −13.71; *P*<.001). Higher starting BMI was associated with greater percentage loss (BMI <25: 5.0%; BMI ≥40: 13.1%).

## Discussion

In this selected cohort of engaged app users, higher food-tracking frequency was associated with greater weight loss and higher rates of 5% or more loss. Outcomes were also more favorable among users with longer consecutive-day tracking streaks. Motivational differences cannot be excluded.

Results are consistent with prior self-monitoring work [5-7], adding adjusted frequency and streak data. Users tracking 2.0 to <3.0 days per week still had a mean weight loss of 7.4%, and 56.5% (271/480) achieved the clinically significant 5% threshold. The app developer conducted the study; the final sample, 1.2% (5132/412,193) of new users after engagement and follow-up filters, was markedly older than excluded goal-setting users (mean age, 42 vs 29 y; 72.8%, 3736/5132 vs 72.2%, 243,048/336,724) women; mean starting BMI (32.4 vs 29.4), limiting generalizability. Residual confounding is likely: motivation, diet quality, physical activity, sleep, and weight loss medications, including glucagon-like peptide-1 (GLP-1) receptor agonists, were not measured; some loss may reflect concurrent pharmacotherapy.

## Supplementary material

10.2196/98144 Multimedia Appendix 1 Association between daily food-tracking frequency and clinically significant weight loss: supplementary tables.

10.2196/98144 Multimedia Appendix 2 A fully anonymized user-level dataset (n = 5132) containing only the outcome indicator (5% or more loss), tracking frequency rounded to 0.1 day/week, age in years, sex, and starting BMI rounded to 0.1, with row order randomized and no identifiers. This file reproduces the primary regression exactly.

## Abbreviations

BMI: body mass index
GLP-1: glucagon-like peptide-1
