# Supplementary material for: Association Between Daily Food-Tracking Frequency and Clinically Significant Weight Loss: Retrospective Cohort Study of 5132 Mobile App Users
Source: J Med Internet Res. 2026 Aug 13;28:e98144. doi: 10.2196/98144 (PMC13472486; doi:10.2196/98144)
Supplement: Supplementary file 1 [file jmir-v28-e98144-s001.docx]

**Multimedia Appendix 2**

Association Between Daily Food-Tracking Frequency and Clinically Significant Weight Loss: Supplementary Tables (Manuscript 98144)

**Table S1. Baseline characteristics by tracking-frequency category (N=5132)**

| **Tracking frequency (days/wk)** | ***n*** | **Mean age, years** | **Women, %** | **Mean starting BMI** |
| --- | --- | --- | --- | --- |
| <2.0 | 475 | 39.7 | 76.4 | 32.3 |
| 2.0 to <3.0 | 480 | 40.1 | 77.9 | 32.7 |
| 3.0 to <4.0 | 540 | 39.3 | 77.6 | 32.3 |
| 4.0 to <5.0 | 642 | 40.7 | 73.7 | 32.0 |
| 5.0 to <6.0 | 770 | 41.4 | 75.5 | 32.1 |
| 6.0 to 7.0 | 2225 | 44.3 | 68.6 | 32.7 |

Categories are nonoverlapping; lower bounds are inclusive, and 7.0 is included in the highest category, as in Table 1 of the manuscript. Age, sex, and starting body mass index (BMI) are the covariates adjusted for in all regression models.

**Table S2. Sensitivity analyses for the association between tracking frequency and 5% or more weight loss**

| **Analysis** | ***n*** | **Adjusted odds ratio (95% CI)** |
| --- | --- | --- |
| Primary: 1 or more food entries per tracked day; 3-SD outlier rule | 5132 | 1.35 (1.31–1.40) |
| 3 or more food entries per tracked day | 5132 | 1.36 (1.31–1.40) |
| 2 or more distinct meal types per tracked day | 5132 | 1.36 (1.31–1.40) |
| More than 800 kcal logged per tracked day | 5132 | 1.27 (1.23–1.31) |
| ±15% weight-change outlier rule | 3933 | 1.32 (1.27–1.37) |

All models adjust for age, sex, and starting BMI; *P* < .001 for all estimates. Tracked-day definitions are applied within the per-user observation window (signup through November 30, 2025). The stricter definitions redefine the exposure on the fixed primary cohort, so n is unchanged across those rows; only the ±15% outlier rule alters cohort membership. The 3-SD outlier rule excludes weight changes beyond 3 SDs of the cohort mean.

**Table S3. Included analytic cohort versus excluded weight-loss goal-setting users**

| **Group** | ***n*** | **Mean age, years** | **Women, %** | **Mean starting BMI** |
| --- | --- | --- | --- | --- |
| Included analytic cohort | 5132 | 42.1 | 72.8 | 32.4 |
| Excluded goal-setting users | 336,814 | 29.1 (a) | 72.2 (b) | 29.4 (c) |

(a) Among excluded users with a plausible recorded date of birth. (b) Among excluded users with recorded sex. (c) Among the 97.4% of excluded users with a plausible weight recorded within 30 days of signup and a plausible height; goal setup collects both, so anthropometric coverage is near complete.
